# Prevalence and correlates of restricted community mobility in a population-based cohort of adults with systemic lupus erythematosus

**DOI:** 10.1136/lupus-2024-001430

**Published:** 2025-03-28

**Authors:** Leila Milanfar, Christopher Barrett Bowling, Courtney Hoge, Amanda Eudy, Patricia Katz, Jinoos Yazdany, Laura Plantinga

**Affiliations:** 1 University of California San Francisco, San Francisco, California, USA; 2 Duke University, Durham, North Carolina, USA; 3 Emory University, Atlanta, Georgia, USA

**Keywords:** prevalence, epidemiology, health-related quality of life, patient reported outcome measures

## Abstract

**Objective:**

Restrictions in community mobility, defined as the frequency of and help required to travel to ‘life-spaces’ (bedroom, home, yard, neighbourhood and town), are associated with poor outcomes among older adults. We aimed to describe and explore factors associated with community mobility among adults with SLE.

**Methods:**

We assessed community mobility cross-sectionally in a population-based SLE cohort (October 2019 to May 2022), using the University of Alabama Birmingham Study of Aging Life-Space Assessment (UAB LSA) (score range, 0–120; higher scores=greater community mobility). Community mobility was considered to be restricted if the individual reported not reaching the neighbourhood life-space or beyond at least weekly and without help. Estimated percentages (95% CIs) with restricted community mobility were assessed with multivariable logistic regression adjusting for demographics and disease activity and damage.

**Results:**

Among 447 participants (91.7% women; 82.6% Black; mean age 46.2; mean UAB LSA score 53.6), 41.6% had restricted community mobility. After adjustment, Black versus White race (43.4% (95% CI 38.5% to 48.2%) vs 24.4% (12.7% to 36.2%)), lowest versus highest educational attainment (51.1% (41.4% to 60.7%) vs 27.2% (20.7% to 33.6%)) and higher versus lower disease activity (55.2% (48.4% to 62.0%) vs 28.5% (22.9% to 34.3%)) were associated with a higher prevalence of restricted community mobility; there were no differences by age, sex or disease damage.

**Conclusion:**

Restricted community mobility was common among adults with SLE, and Black race, lower education and high disease activity were associated with more restricted community mobility. Further research to understand the association of community mobility with outcomes and implement strategies to improve community mobility in people with SLE is warranted.

**WHAT IS ALREADY KNOWN ON THIS TOPIC**

- Among older adults, restricted community mobility, defined by the frequency of and help required for travelling to ‘life-spaces’ (bedroom, home, yard, neighbourhood and town), is associated with reduced quality of life and greater risk of falls, hospitalisation and mortality. However, little is known about community mobility among the ageing population with SLE.

**WHAT THIS STUDY ADDS**

- We found that restricted community mobility was common, with 42% not independently travelling to their neighbourhood life-space at least weekly.
- It was more prevalent among those who were Black, had lower educational attainment and reported higher disease activity; however, there was no difference in restricted community mobility by age in our cohort.

**HOW THIS STUDY MIGHT AFFECT RESEARCH, PRACTICE OR POLICY**

- While further research is needed to understand the association of restricted community mobility with outcomes among those with SLE and to target strategies to support community mobility in this population, healthcare providers should recognise that restricted community mobility is common in SLE and should consider individualised interventions that address restricted community mobility, such as physical or occupational therapy, support services or better control of SLE.

## Introduction

 Community mobility and social participation are critical aspects of overall health and quality of life for older adults. The University of Alabama at Birmingham (UAB) Life-Space Assessment (LSA) measures these concepts by integrating a person’s independence and frequency of movement to different ‘life-spaces’ (within and outside their bedroom, home, yard, neighbourhood and town) into a score. Among older adults, lower LSA scores are associated with physical limitations, limitations in activities of daily living, frailty, poor mental health and increased risk of cognitive decline among older adults.^1^ This score has also been identified as a predictor of hospital admissions, falls, mortality and quality of life in older adults.^1^ Community mobility has been assessed in older adults and in those with various chronic diseases and conditions,^2^,^10^ but it remains understudied in rheumatic diseases, including SLE, where the social isolation resulting from restrictions in community mobility is prevalent and associated with poor psychosocial outcomes.^11 12^

We previously administered the UAB LSA in a pilot study of individuals with SLE.^13^ Interestingly, our results indicated that scores were low overall (mean, 54) and that younger patients with SLE reported lower LSA scores than older patients with SLE. These findings stand in contrast to findings from the UAB Study of Ageing older adult population, in which older age was associated with lower LSA scores. Building on our pilot work, here we aim to provide an epidemiological description of community mobility in the larger Approaches to Positive, Patient-centred Experiences of Ageing with Lupus (APPEAL) study, as well as to examine sociodemographic and clinical factors associated with community mobility in people with SLE.

## Methods

### Study population and data sources

APPEAL participants were recruited from the ongoing, population-based Georgians Organised Against Lupus (GOAL) cohort.^14^ Briefly, GOAL participants are adults (≥18 years) living in metropolitan Atlanta who have SLE, defined as ≥4 revised American College of Rheumatology (ACR) criteria^15^ or 3 ACR criteria plus a diagnosis of SLE by an attending board-certified rheumatologist. For APPEAL, detailed assessments of physical and cognitive functioning across multiple domains, including community mobility, were obtained during a single study visit. Inclusion criteria for the APPEAL study were: ability to speak English, sufficient vision and hearing to undergo study testing and currently living in Georgia.

A total of 451 participants completed a single APPEAL study visit (206 in-person and 245 remote visits^16^) between October 2019 and May 2022. For this analysis, we excluded participants whose overall survey response patterns were potentially invalid due to lack of variation in responses to instruments with reverse coding (n=4), leaving n=447 participants for analyses. Cross-sectional study data were obtained from performance tests and self-administered questionnaires during the APPEAL visit and from annual GOAL surveys closest to the APPEAL visit date.

### Patient and public involvement

Patients were involved in the design and conduct of this research. We used feedback from our pilot study of patients^13^ to create our initial protocol; patient participant feedback was also used to modify the protocol as needed throughout the course of this study. Patient burden was carefully considered in the number and order of measures assessed; patients were able to skip assessments and take breaks as needed. Finally, GOAL participants are informed of study results through regular study newsletters suitable for a non-specialist audience.

### Variables

#### Community mobility

**Figure 1:**
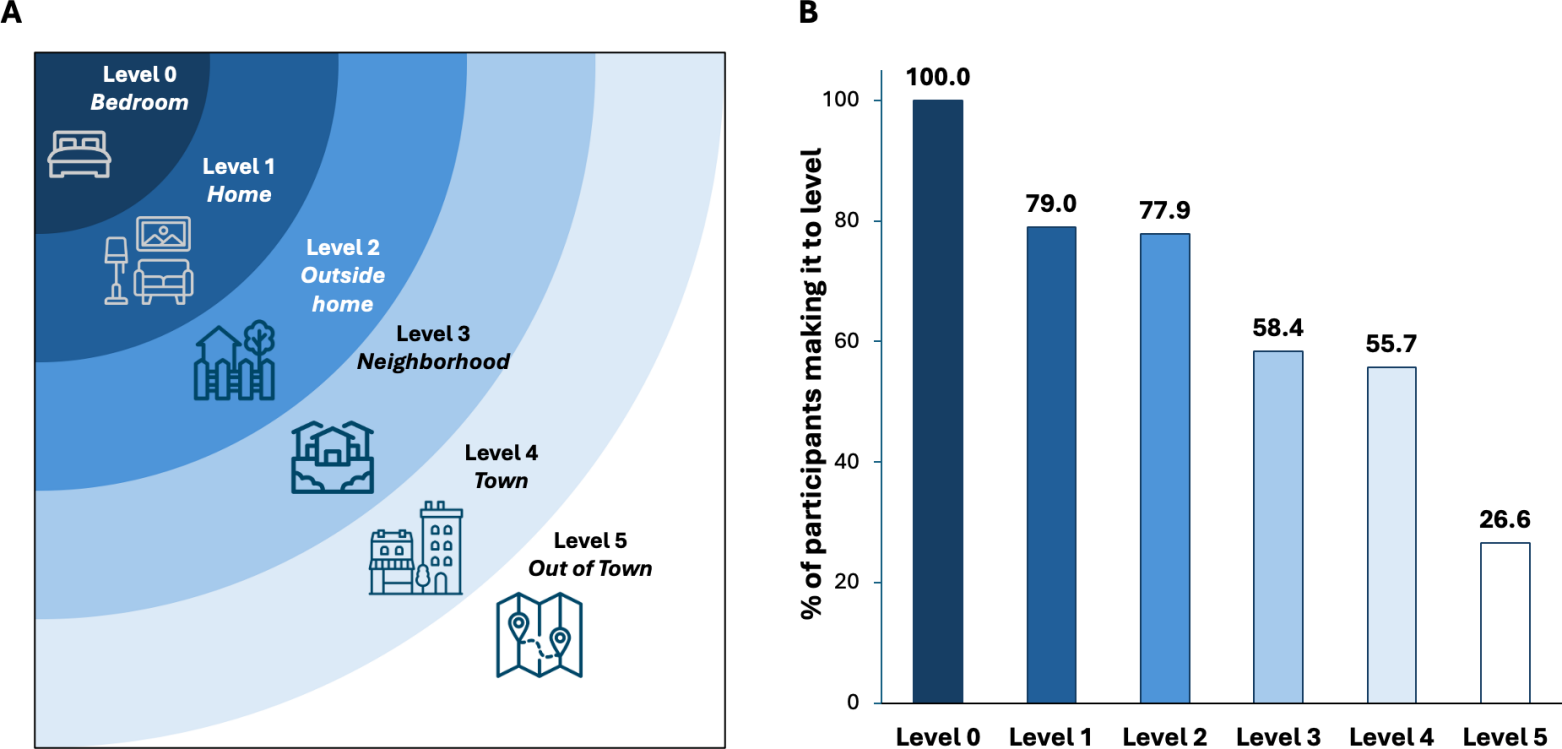
Life-space levels (**A**) and percentage of participants reaching these levels at least weekly and without assistance from a device or person (**B**). Adapted from Peel *et al*.^18^ Icons courtesy of The Noun Project under a Creative Commons licence (Artists: bed, yode tiva; home: Yogi Aprelliyanto; outside home, Katerine Melina; neighbourhood: Eklip Studio; town, Made by Made; out of town, IYIKON).

We used the UAB LSA^17 18^ to capture community mobility and social participation, which measures how far respondents have gone (life-space levels: bedroom (life-space level 0), other rooms in the home (life-space level 1), outside the home (life-space level 2), neighbourhood (life-space level 3), town (life-space level 4) and out of town (life-space level 5); figure 1A) over the past 4 weeks. The score also captures how often and with how much help respondents went to these life-space levels. Overall scores are then calculated by summing the scores for each of the five levels and range from 0 to 120, with higher scores representing greater community mobility (online supplemental table S1). Our primary outcome was restricted community mobility, which we defined as the inability to reach the neighbourhood level at least weekly and without assistance from either a device or person (restricted vs not). This definition was chosen based on the likely effect of this level of restriction on access to food and other necessary items, work, medical care and other services and social opportunities.

#### Other variables

We examined variables that were potentially associated with community mobility. Sociodemographic variables, including age, sex (at birth), race, ethnicity and education, were self-reported by the participant via the NIH Toolbox from a fixed set of categories. Race was categorised as Black (single or multiple race), White and other. Education was the highest level attained and categorised as high school graduate/equivalency or lower, some college/associate degree and college graduate or higher. Current working status was assessed with the Work Productivity and Activity Impairment Questionnaire: General Health V.2.0.^19^,^21^ Current income (categorised as <US$30 000, US$30 000–US$69 999 and US$70 000+, based on the distribution of income in our cohort) and receipt of Social Security disability benefits were self-reported at the closest GOAL assessment. SLE-related factors included measures of disease duration, activity and damage. Disease duration was self-reported during the GOAL assessment and adjusted for the date of the APPEAL assessment. Current SLE activity was assessed during the APPEAL visit via the Systematic Lupus Activity Questionnaire (SLAQ; range 0–44; higher scores indicating greater SLE-related disease activity).^22^ The Brief Index of Lupus Damage (BILD) score (range, 0–46; higher scores indicate greater cumulative SLE-related organ damage)^23 24^ closest to the APPEAL visit was obtained from linked GOAL data; cumulative system damage was also defined by the BILD. Current steroid use was self-reported by the participant at the study visit. Other factors included body mass index (BMI), which was calculated from measured (in-person visits) or reported (remote visits) weight and height as (weight in kg)/(height in m^2^); obesity was defined as BMI ≥30 mg/kg^2^. Physical activity was assessed with the International Physical Activity Questionnaire-Short Form.^25^ Depressive symptoms were assessed via the validated 8-item Patient-Reported Outcomes Measurement Information System Depression Short Form-8a^26 27^ and reported as T-scores (where 50=mean score and 10=1 SD). Finally, perceived stress was assessed using the 10-item Perceived Stress Scale (range 0–40; higher scores indicate greater perceived stress, with scores of ≥20 considered high levels of stress).^28^ The pandemic era of visit was defined as prepandemic (October 2019–early March 2020), early pandemic (October 2020–April 2021; there were no study visits between early March 2020 and October 2020) and late pandemic (May 2021–May 2022).

### Statistical analysis

Descriptive statistics were used to describe the characteristics of the overall study population and the distribution of LSA scores among APPEAL participants. Multivariable logistic regression with marginal estimation was used to estimate associations between various participant characteristics and restricted community mobility; marginal estimates were expressed as percentages. Confounders were defined a priori (age, sex, disease damage) and by their associations with the characteristics and LSA score (race, education and work status); variables that were highly correlated with other confounders (disability status, income) were not included. In sensitivity analyses, we examined: (1) models with additional adjustment for depressive symptoms, which were left out of primary models due to their potential mediating role; (2) models not including work status, which may not be a confounder; (3) models looking at restrictions at any level versus no restrictions travelling out of town without assistance and (4) models using continuous LSA scores instead of dichotomised community mobility. Because of the possibility of the effects of the pandemic on community mobility, we additionally examined: (5) models stratified by type of visit (in-person vs remote) and by pandemic era. Complete case analysis was used, and the statistical significance threshold was set at 0.05. Analyses were performed using Stata V.18.5 (College Station, Texas, USA).

## Results

### Characteristics of study participants

Study participants had a mean age of 46.2 years; 40.9% were ≥50 years of age (table 1). Most were female (91.7%), Black (82.6%) and non-Hispanic (94.4%). About one-quarter (23.0%) had an education level of high school or less, and 35.5% had an annual household income of <US$30 000. About half (52.9%) were not working at the time of the encounter and nearly half (45.6%) reported receiving Social Security disability support. The median duration of SLE was 14.8 years, and median SLAQ and BILD scores were 11 and 2, respectively. Participants had a mean BMI of 30.1 kg/m² (47.3% obese) and 73.9% reported low physical activity. About half were currently taking steroids (42.8%) or immunosuppressive medications (46.3%), while 71.8% were taking hydroxychloroquine. The mean physical functioning T-score was 42.8, and the mean depressive symptoms T-score was 48.2. Perceived stress scores averaged 15.2. Over half of the participants’ study visits were performed remotely (54.4%) and during the late pandemic era (1 May 2021 to 12 May 2022; 55.9%) (table 1).

**Table 1:** Selected characteristics of study participants* with SLE

Characteristic	Mean (SD), median (IQR) or n (%)
Sociodemographic	
Mean (SD) age in years	46.2 (11.8)
Age category (in years), n (%)	
18–34	91 (20.4%)
35–49	173 (38.7%)
≥50	183 (40.9%)
Sex,† n (%)	
Female	410 (91.7%)
Male	37 (8.3%)
Race, n (%)	
Black	369 (82.6%)
Other	27 (6.0%)
White	51 (11.4%)
Hispanic ethnicity, n (%)	
Yes	25 (5.6%)
No	421 (94.4%)
Education	
High school degree or lower	103 (23.0%)
Some college/associates degree	170 (38.0%)
College graduate or higher	174 (38.9%)
Currently working, n (%)	
Yes	205 (47.1%)
No	230 (52.9%)
Current household income (US$), n (%)	
<30 000	154 (35.5%)
30 000–69 999	172 (39.6%)
70 000+	108 (24.9%)
Social security disability support,‡ n (%)	
Yes	202 (45.6%)
No	241 (54.4%)
Clinical	
Median (IQR) disease duration, years‡	14.8 (9.2, 22.3)
Median (IQR) SLAQ score	11.0 (6.0, 16.0)
Median (IQR) BILD score‡	2.0 (1.0, 4.0)
Currently taking steroids, n (%)	
Yes	191 (42.8%)
No	255 (57.2%)
Currently taking hydroxychloroquine, n (%)	
Yes	320 (71.8%)
No	126 (28.3%)
Currently taking immunosuppressive medication,‡‡ n (%)	
Yes	207 (46.3%)
No	240 (53.7%)
Mean (SD) BMI, kg/m^2^	30.1 (8.2)
Obesity, n (%)	
Yes	206 (47.3%)
No	230 (52.7%)
Physical activity level,§ n (%)	
Low	325 (73.9%)
Moderate	67 (15.2%)
High	48 (10.9%)
Mean (SD) physical functioning T-score¶	42.8 (37.3–47.9)
Mean (SD) depressive symptoms T-score**	48.2 (37.1–55.3)
Mean (SD) perceived stress score††	15.2 (7.2)
Other	
Type of visit, n (%)	
In-person	204 (45.6%)
Remote	243 (54.4%)
Era of visit, n (%)	
Prepandemic (10 August 2019 to 3 October 2020)	50 (11.2%)
Early pandemic (10 June 2020 to 30 April 2021)	147 (32.9%)
Late pandemic (1 May 2021 to 12 May 2022)	250 (55.9%)

* N=447 for all except: ethnicity (*N*n=446), employment (*N*n=435), income (*N*n=434), disability support (*N*n=443), disease duration (*N*n=446), SLAQ (*N*n=428), BMI/obesity (*N*n=436), physical activity (*N*n=440), steroids (*N*n=446), depressive symptoms (*N*n=430), perceived stress (*N*n=413).

† Represents sex assigned at birth.

‡ From the closest Georgians Organised Against Lupus (GOAL; parent study) assessment.

§ From the International Physical Activity Questionnaire – -Short Form.

¶ From the (PROMIS) Physical Functioning 10-item Short Form (a) (higher T-scores representing better self-reported physical functioning).

** From the PROMIS Depression Short Form-8a.

†† From Cohen’s 10-item Perceived Stress Scale (range, 0–40; higher scores representing greater perceived stress).

‡‡ Methotrexate, cyclophosphamide, ciclosporincyclosporine, mycophenolic acid, azathioprine, belimumab, rituximab, or antitumour necrosis factor-TNF.

BILDBrief Index of Lupus Damage (range 0–46; 46 is maximum damage)BMIbody mass indexPROMISPatient-Reported Outcomes Measurement Information SystemSLAQSystemic Lupus Activity Questionnaire (range 0–47; 47 is maximum activity)

### Community mobility

The mean (SD) LSA score was 53.6 (34.1), with a median (IQR) of 58.0 (24.0–80.0) (online supplemental figure S1). Scores were higher in White versus Black participants (67.2 vs 51.7; p=0.01) and those with higher versus lower SLAQ scores (61.5 vs 43.5; p<0.001), but did not differ by age, sex, BILD scores, visit type or phase of pandemic (online supplemental figure S2). Most participants (79.0%) reached life-space level 1 (beyond the bedroom), 77.9% reached life-space level 2 (outside the home), 58.4% reached life-space level 3 (into the neighbourhood), 55.7% reached life-space level 4 (into the town) and 26.6% reached life-space level 5 (outside of town) at least weekly and without any assistance from a device or a person (figure 1B). By our primary definition, 41.6% had restricted community mobility.

### Associations of restricted community mobility with participant characteristics

Prevalence of restriction in community mobility was higher in Black (44.2%) vs White (23.5%; p=0.006) participants, but this difference was not statistically significant after adjustment for socioeconomic factors (table 2). Lower educational attainment was associated with a higher prevalence of restriction in community mobility, with 53.4% and 49.4% of participants who completed high school or less and some college having restrictions, vs 27.0% of participants with college degrees or higher (p<0.001 for both); these differences remained statistically significant with full adjustment. Similarly, those who were not currently working versus currently working were more likely to have restricted community mobility (50.0% vs 30.2%; p<0.001), and this difference remained statistically significant after full adjustment. Participants with the lowest household annual income (<US$30 000) were more likely to have restricted community mobility than those with income of US$70 000 or more (57.8% vs 29.6%; p<0.001), as were those receiving disability support versus not (53.5% vs 31.5 %, p<0.001), but these differences did not persist with adjustment for other socioeconomic factors. Participants with higher versus lower SLAQ scores were more likely to have restricted community mobility (56.1% vs 29.7%; p<0.001) and this difference persisted regardless of adjustment (table 2). Those with moderate or high physical activity were less likely than those with low physical activity to have restricted community mobility (23.5% vs 47.4%; p<0.001), and this difference persisted with adjustment (table 2). Those with higher versus lower scores for depressive symptoms (49.2% vs 35.2%; p=0.003) and perceived stress (49.2% vs 33.5%; p=0.001) were more likely to have restricted community mobility, even after adjustment for demographic and socioeconomic factors; however, neither difference was statistically significant after adjustment for clinical factors. While disease damage (higher vs lower BILD score) and current steroid use were both associated with higher percentages of individuals with restricted community mobility, these differences were not statistically significant, regardless of adjustment. Finally, there were no differences in percentages of participants with restricted community mobility by sex, obesity or age (table 2); further subdividing age, we found that the percentages with restricted community mobility were lowest in those who were 20–29 years of age (32.9%) vs 45.0%, 44.1% and 39.9% in those aged 30–39, 40–49 and ≥50 years, but the difference was not statistically significant.

**Table 2:** Percentages of individuals with restricted community mobility by selected participant characteristics

Characteristic	Percentages with restricted mobility (95% CI)			
	Unadjusted	Adjusted for demographics*	Additionally adjusted for socioeconomic factors*	Additionally adjusted for clinical factors*
Age (years)				
18–34	41.8 (31.6 to 51.9)	41.8 (31.7 to 51.8)	40.2 (30.2 to 50.2)	41.0 (31.1 to 50.8)
35–49	43.3 (36.0 to 51.9)	43.4 (36.0 to 50.7)	41.9 (34.8 to 49.1)	40.4 (33.4 to 47.3)
≥50 (ref.)	39.9 (32.8 to 47.0)	39.9 (32.9 to 46.9)	39.8 (33.0 to 46.6)	41.2 (34.5 to 48.1)
Sex				
Women (ref.)	41.7 (36.9 to 46.5)	41.7 (37.0 to 46.4)	40.8 (36.2 to 45.5)	40.7 (36.2 to 45.3)
Men	40.5 (24.7 to 56.4)	40.5 (24.8 to 56.2)	38.9 (23.6 to 54.1)	42.4 (26.7 to 58.1)
Race				
Black (ref.)	44.2 (39.4 to 49.5)	44.2 (39.4 to 49.5)	43.3 (38.4 to 48.3)	43.4 (38.5 to 48.2)
Other	37.0 (18.8 to 55.3)	37.0 (18.8 to 55.3)	38.5 (20.5 to 56.5)	36.0 (18.4 to 53.6)
White	23.5 (11.9 to 35.2)†	23.5 (11.9 to 35.2)†	22.4 (11.1 to 33.8)	24.4 (12.7 to 36.2)
Education				
≤High school graduate	53.4 (43.8 to 63.0)‡	53.4 (43.8 to 63.0)‡	52.0 (42.3 to 61.7)†	51.1 (41.4 to 60.7)†
Some college	49.4 (41.9 to 56.9)‡	49.4 (41.9 to 56.9)‡	48.5 (41.0 to 56.0)†	48.8 (41.4 to 56.1)†
≥College graduate (ref.)	27.0 (20.4 to 33.6)	27.0 (20.5 to 33.6)	26.5 (19.9 to 33.0)	27.2 (20.7 to 33.6)
Currently working				
Yes	30.2 (24.0 to 36.5)‡	30.2 (24.0 to 36.5)‡	30.2 (24.1 to 36.5)†	30.5 (24.4 to 36.5)
No (ref.)	50.0 (43.5 to 56.5)	50.0 (43.6 to 56.4)	50.0 (43.7 to 56.3)	50.2 (44.0 to 56.5)
Household annual income (US$)§				
<30 000	57.8 (50.0 to 65.6)‡	57.8 (50.0 to 65.6)†	55.7 (47.9 to 63.7)	55.3 (47.5 to 63.1)
30 000–69 999	35.5 (28.3 to 42.6)	35.5 (28.3 to 42.6)	35.3 (28.3 to 42.3)	36.1 (29.2 to 43.0)
≥70 000 (ref.)	29.6 (21.0 to 38.2)	29.6 (21.1 to 38.2)	29.5 (21.1 to 38.0)	30.6 (22.1 to 39.1)
Receiving disability support§				
Yes	53.5 (46.6 to 60.3)‡	53.5 (46.6 to 60.3)‡	52.3 (45.4 to 59.2)	52.9 (46.1 to 59.7)
No (ref.)	31.5 (25.7 to 37.4)	31.5 (25.7 to 37.4)	31.2 (25.5 to 36.9)	31.0 (25.3 to 36.7)
Disease activity				
SLAQ score <median (ref.)	29.7 (23.9 to 35.6)	29.7 (23.9 to 35.6)	28.6 (22.8 to 34.3)	28.5 (22.9 to 34.3)
SLAQ score ≥median	56.1 (49.2 to 63.1)‡	56.1 (49.2 to 63.0)‡	55.2 (48.4 to 62.0)‡	55.2 (48.4 to 62.0)‡
Disease damage				
BILD score <median (ref.)	39.5 (33.7 to 45.3)	39.5 (33.7 to 45.3)	38.9 (33.3 to 44.4)	38.3 (32.8 to 43.8)
BILD score ≥median	45.0 (37.6 to 52.5)	45.0 (37.6 to 52.5)	43.6 (36.3 to 50.9)	45.0 (37.8 to 52.2)
Current steroids				
Yes (ref.)	46.6 (39.5 to 53.7)	46.6 (39.6 to 53.6)	45.2 (38.3 to 52.0)	45.7 (38.7 to 52.6)
No	37.6 (31.7 to 43.6)	37.6 (31.8 to 43.5)	37.1 (31.3 to 42.9)	37.2 (31.6 to 42.8)
Obese				
Yes	40.8 (34.1 to 47.5)	40.8 (34.1 to 47.4)	39.8 (33.3 to 46.3)	40.0 (33.6 to 46.4)
No (ref.)	42.6 (36.2 to 49.0)	42.6 (36.2 to 48.9)	41.7 (35.5 to 47.9)	42.2 (36.1 to 48.3)
Physical activity				
Moderate-to-high	23.5 (15.7 to 31.2)	23.5 (15.8 to 31.2)	23.0 (15.5 to 30.6)	22.4 (14.9 to 29.9)
Low (ref.)	47.4 (42.0 to 52.8)	47.4 (42.0 to 52.8)	46.5 (41.2 to 51.8)	47.0 (41.8 to 52.3)
Depressive symptoms				
PROMIS score <median (ref.)	35.2 (29.1 to 41.3)	35.2 (29.1 to 41.3)	33.9 (28.0 to 39.8)	33.6 (27.8 to 39.5)
PROMIS score ≥median	49.2 (42.3 to 56.2)†	49.2 (42.4 to 56.1)†	48.4 (41.7 to 55.2)†	49.4 (42.6 to 56.3)
Perceived stress				
PSS score <median (ref.)	33.5 (27.3 to 39.7)	33.5 (27.3 to 39.6)	33.0 (27.0 to 39.0)	33.2 (27.2 to 39.1)
PSS score ≥median	49.2 (42.1 to 56.3)†	49.2 (42.1 to 56.3)†	48.1 (41.2 to 55.0)†	47.8 (40.9 to 54.6)

* Demographics: continuous age, sex, race; socioeconomic: education and work status; clinical characteristics: continuous SLAQ and BILD scores.

† P<0.05 vs. referent, from logistic regression.

‡ P<0.001 vs. referent, from logistic regression.

§ From the closest Georgians Organised Against Lupus (GOAL; parent study) assessment.

BILD, Brief Index of Lupus Damage; BMI, body mass index; PROMIS, Patient-Reported Outcomes Measurement Information System (Depression Short Form-8a); PSS, Perceived Stress Scaleref.referenceSLAQ, Systemic Lupus Activity Questionnaire

In sensitivity analyses, we found that neither additional adjustment for depressive symptom scores nor removal of the work variable from the models substantially changed the observed primary results (online supplemental table S2). When we used a stricter definition of restricted community mobility (any restriction at any life-space level), the percentages of individuals with SLE with restricted community mobility were much higher, but the patterns by characteristic were generally similar to those seen in the primary results. However, the difference in percentage with restricted community mobility by disease activity (higher vs lower SLAQ score, 79.7% vs 68.3%) was not statistically significant with this definition (online supplemental table S2). When we used the continuous LSA score as the outcome, fully adjusted differences in mean scores followed similar patterns to the primary results (online supplemental table S2). Finally, we examined whether there was effect modification in any of the observed associations by mode of the visit (in-person vs remote) or by the era (prepandemic, early pandemic and late pandemic; online supplemental table S3). We found that patterns in percentages with restricted community mobility were generally similar across subgroups and that none of the interaction terms were statistically significant (online supplemental table S3).

## Discussion

In this cross-sectional study of a population-based cohort of adults with SLE, we found a high prevalence of restricted community mobility. Despite an average age of only 46 years, 42% of the cohort did not independently travel into their neighbourhood or areas beyond at least weekly. Additionally, participants who were Black, who had lower educational attainment and income and who were not currently employed all had a higher prevalence of restricted community mobility relative to their counterparts, even after adjustment for potential confounders; those with higher disease activity and higher scores for depressive symptoms also had a higher prevalence of restricted community mobility. Notably, the lack of association between LSA score and age suggests that factors other than ageing may be driving community mobility restrictions in people with SLE.

Overall, LSA scores in our cohort of people with SLE demonstrate more community mobility restrictions compared with previously published estimates in other populations. We found that the mean LSA score in our cohort (mean age, 46 years) of individuals with SLE was 54 years, which was substantially lower than the mean scores of 63 (mean age, 75 years)^17^ and 64 (³65 years only)^18^ reported among cohorts of older adults. Our estimates also suggest more community mobility restrictions in SLE compared with older populations with other serious chronic conditions. For example, the mean LSA score in a cohort of participants with Parkinson’s disease (mean age, 71 years) was 64.^9^ Mean LSA scores in cohorts of participants with cardiovascular disease (mean LSA=74, mean age=79),^2^ kidney disease (mean LSA=58, mean age=78),^6^ peripheral artery disease (mean LSA=59, mean age=76),^4^ chronic obstructive pulmonary disease (mean LSA=70, mean age=66)^7^ and progressive multiple sclerosis (mean LSA=80, mean age=64)^10^ were also higher than those of our participants with SLE. Although scores among populations with poststroke sequelae (mean LSA=48, mean age=74)^3^ and heart failure (mean LSA=50, mean age=77)^5^ were lower than those among our cohort of participants with SLE, these populations were, on average, also much older than our cohort. Overall, these findings suggest that restricted community mobility is both prevalent and potentially more severe in individuals with SLE than in those with other chronic conditions. The disproportionate effect of SLE on community mobility, relative to other chronic diseases, may be attributable to its widespread and heterogeneous systemic effects, such as: symptoms associated with joint and muscle inflammation, which can cause difficulty with ambulation and travel; dermatological manifestations, which can lead to both restrictions in outdoor activities and anxiety about sun exposure; renal, cardiac and pulmonary damage, which can cause activity-limiting symptoms including shortness of breath, lower extremity oedema and pain; fatigue, which can cause individuals to limit activities and exertion; and depression, which can cause anhedonia and be associated with many symptoms, including pain and fatigue.

Interestingly, in our study, we saw no association between LSA score and age. This finding stands in contrast to findings in individuals without SLE, among whom older age was associated with lower LSA score,^1^ and also to findings from our pilot study, in which younger age was associated with lower LSA score.^13^ One possible reason for a lack of association of LSA scores with age may include survivorship bias in this prevalent cohort, due to the younger patients with more severe disease (and, likely, more restricted community mobility) having shorter survival. This bias could have masked an association between older age and higher LSA score in those with SLE. Of course, it is also possible that, in the setting of a heterogeneous and intermittent disease like SLE, older age does not predict more restricted community mobility. Instead, restricted community mobility may be more closely tied to self-imposed social isolation due to psychosocial distress and perceived stigma surrounding lupus.^11 12^ Further longitudinal studies of the associations between age and LSA score in participants with newly diagnosed SLE are warranted.

Similarly, although prior studies of older adults consistently found that female sex was associated with lower LSA scores,^1 18 29^ we found no differences in community mobility between women and men with SLE. Several studies suggest that SLE can be more severe in men, which might explain our findings. However, it is also possible that this reflects an underpowered analysis, as only ~8% of our participants were male. Again, further study of potential sex differences in community mobility in the setting of SLE is warranted.

We also found several patterns that mirror those seen in older adults. For example, previous studies of older adults^18 29^ showed that Black individuals have more restricted community mobility than White individuals. Similarly, we found that Black participants from our cohort reported a substantially higher prevalence of community mobility restriction than White participants (44% vs 24%). Systematic racism and inequity have been posited to explain the associations between race and community mobility, with racial segregation, disparities in transportation or housing access and in education or employment opportunities, inequitable infrastructure development and bias in criminal justice possibly playing important roles.^30 31^ Neighbourhood disadvantage status and individual-level poverty may also contribute to constricted community mobility.^32^ Metropolitan Atlanta is a highly racially segregated metropolitan area,^33^ and it is also the metropolitan area with the highest degree of income inequality in the USA.^34^

One important limitation of our study is that we did not have access to geospatial data to examine these contextual factors, nor did we have data on participants’ perceptions of these phenomena. A critical next step would be to examine contextual factors associated with community mobility restriction and, importantly, subsequent outcomes among individuals with SLE. For example, those in more deprived or rural areas may have to travel further (and therefore navigate to more distant life-spaces) due to fewer resources in their immediate life-space. The built environment could also contribute: for example, accessible homes and services; clean, unobstructed and shaded sidewalks and/or an extensive, affordable and easy-to-use public transportation system could all lead to less restricted community mobility. Crime rates could also play a role, as individuals who perceive their proximal life-spaces to be unsafe may travel to them or beyond less frequently. Future studies examining these factors are warranted.

The interplay between depressive symptoms and community mobility has been previously described in non-SLE populations.^1 8 35^ Lech *et al*^36^ demonstrated that community mobility restriction in older adults was associated with clinically significant depression and that, in fact, utilisation of mental healthcare services appeared to be associated with less community mobility restriction. In our SLE population, we found that a higher burden of depressive symptoms was associated with greater restriction in community mobility and lower LSA scores, although this association did not persist after adjustment for disease activity, which may reflect the correlation between disease activity and depressive symptoms. Further study, including longitudinal data, would be needed to assess the temporal and causal relationships between depressive symptoms and community mobility.

Because community mobility has been rarely studied in SLE, little is known about the associations between measures of lupus disease activity, cumulative damage and community mobility. In our pilot study, higher SLAQ scores were associated with lower community mobility, while no association between BILD scores and LSA scores was noted.^13^ Here, we corroborated these findings. Increased disease activity may be associated with decreased community mobility through multiple mechanisms, including decreased physical mobility (due to joint pain, fatigue or other physical manifestations of SLE), reduced cognitive function and/or increased mental health burden. However, it is less readily apparent why cumulative disease damage does not additionally correlate with decreased community mobility. One possible limitation regarding the BILD score in our cohort is that BILD reflects quite severe damage; thus, most individuals in our cohort have low BILD scores. We were, therefore, underpowered to examine the impact of higher levels of disease damage.

In addition to the limitations regarding survivorship bias and lack of data on contextual factors, discussed above, there are other limitations to our study that deserve mention. First, the COVID-19 pandemic occurred during our recruitment, which resulted in a mixture of in-person and remote visits; the individuals completing these two types of visits may have differed systematically in unmeasured ways. Furthermore, the era of the pandemic itself (prepandemic, as well as early vs late pandemic) may have affected community mobility due to various restrictions and individual risk tolerance. However, in our sensitivity analyses, we found no substantial differences in observed associations with community mobility by either visit modality or pandemic era, suggesting that any bias due to these factors was minimal. Second, the use of a cross-sectional design limits causal inference. Future studies of community mobility should include longitudinal studies assessing associations of characteristics with subsequent community mobility. Third, we had limited power for analyses by certain subgroups of interest, such as by ethnicity and by more granular younger age groups, who may face unique challenges in the transition from paediatric to adult care. Finally, as with any observational study, there remains the possibility of residual confounding by unknown or unmeasured factors.

In summary, we found that a high prevalence of restricted community mobility among adults with SLE and identified several factors, including Black race, lower educational attainment and higher disease activity, that were associated with a higher prevalence of these restrictions. Given that restricted community mobility likely limits their social participation and is a risk factor for a variety of poor outcomes across many chronic conditions, healthcare providers should recognise that restricted community mobility is common in SLE and should consider interventions that address restricted community mobility. Such interventions might include addressing physical symptoms (*eg,* disease activity, pain, fatigue, deconditioning), identifying mental health factors (*eg,* depression, social isolation), and referring for services that address underlying causes of restricted community mobility or mitigate barriers to community mobility (*eg,* transportation services). More research examining longitudinal associations of characteristics with community mobility and of community mobility with subsequent health outcomes (including, but not limited to, quality of life, falls, institutionalisation, and mortality) in SLE is needed. Such studies could help providers overcome assumptions based on a patient’s age or ability to attend a medical visit and better understand the impact of SLE on patients’ community mobility and social participation. This information could also help identify modifiable factors — such as disease activity, depressive symptoms, or limited physical mobility — that could be better managed or supported. Further, this assessment could also inform patient-centred, goal-oriented communication in SLE, with the ultimate goal of improving the health and quality of life for the diverse population of people with SLE.

## supplementary material

10.1136/lupus-2024-001430 online supplemental file 1

## Data Availability

Data are available on reasonable request.
