# Supplementary material for: Prevalence and correlates of restricted community mobility in a population-based cohort of adults with systemic lupus erythematosus
Source: Lupus Sci Med. 2025 Mar 28;12(1):e001430. doi: 10.1136/lupus-2024-001430 (PMC11956307; doi:10.1136/lupus-2024-001430)
Supplement: online supplemental file 1 [file lupus-12-1-s001.pdf]

Supplement: Milanfar *et al*., Prevalence and Correlates of Restricted Community Mobility in a Population-based Cohort of Adults with Systemic Lupus Erythematosus

Table of Contents

Table S1. Items and scoring for our adapted version of the University of Alabama Birmingham Life-Space Assessment 2

Figure S1. Distribution of University of Alabama Birmingham Life-Space Assessment scores among study participants. 3

Figure S2. University of Alabama Birmingham Life-Space Assessment scores by selected participant characteristics........ 4

Table S2. Sensitivity analyses: associations of selected participant characteristics with additional adjustment for depressive symptoms; with the cutoff defined as any restriction in community mobility; and using continuous scores 5

Table S3. Sensitivity analyses: associations of selected participant characteristics, stratified by type and era of study visit 7

Table S1. Items and scoring for our adapted version of the University of Alabama Birmingham Life-Space Assessment

	**Life-Space Level**	**Frequency**	**Help**
**Item**	During the past 4 weeks, *with the exception of this study visit*, have you been to…	How often did you get there?	Did you need help from an aid or equipment?<br>Did you need the help of another person?
**Scoring**	1=Yes<br>0=No	1=<1 time per week<br>2=1-3 times per week<br>3=4-6 times per week<br>4=daily	1=Help from another person<br>1.5=Help from equipment only<br>2=No help
**Life-Space Level Scores:**	***Level* × *Frequency* × *Help***		
1	…other rooms of your home besides the room where you sleep?		
2	…an area outside your home such as your porch, deck/patio, hallway of apartment building, garage, yard/driveway?		
3	…places in your neighborhood other than yard or apartment building?		
4	…places outside your neighborhood but within your town?		
5	…places outside your town?		
**Total Score**	**Sum of all Life-Space Level Scores**		

Figure S1. Distribution of University of Alabama Birmingham Life-Space Assessment scores among study participants.

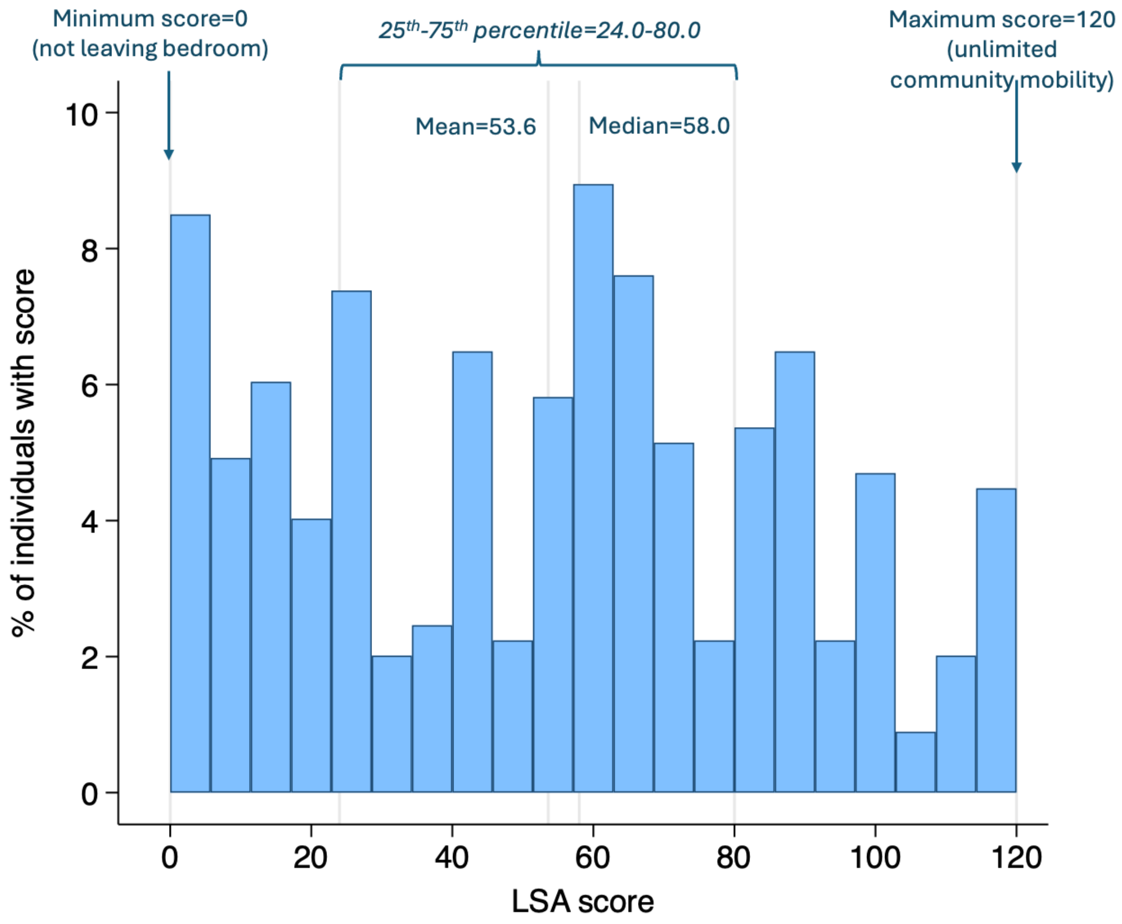

Figure S2. University of Alabama Birmingham Life-Space Assessment scores by selected participant characteristics.

P-values from t test or ANOVA, as appropriate; P>0.15 except where indicated. LSA, Life-Space Assessment; SLAQ, Systemic Lupus Activity Questionnaire; BILD, Brief Index of Lupus Damage.

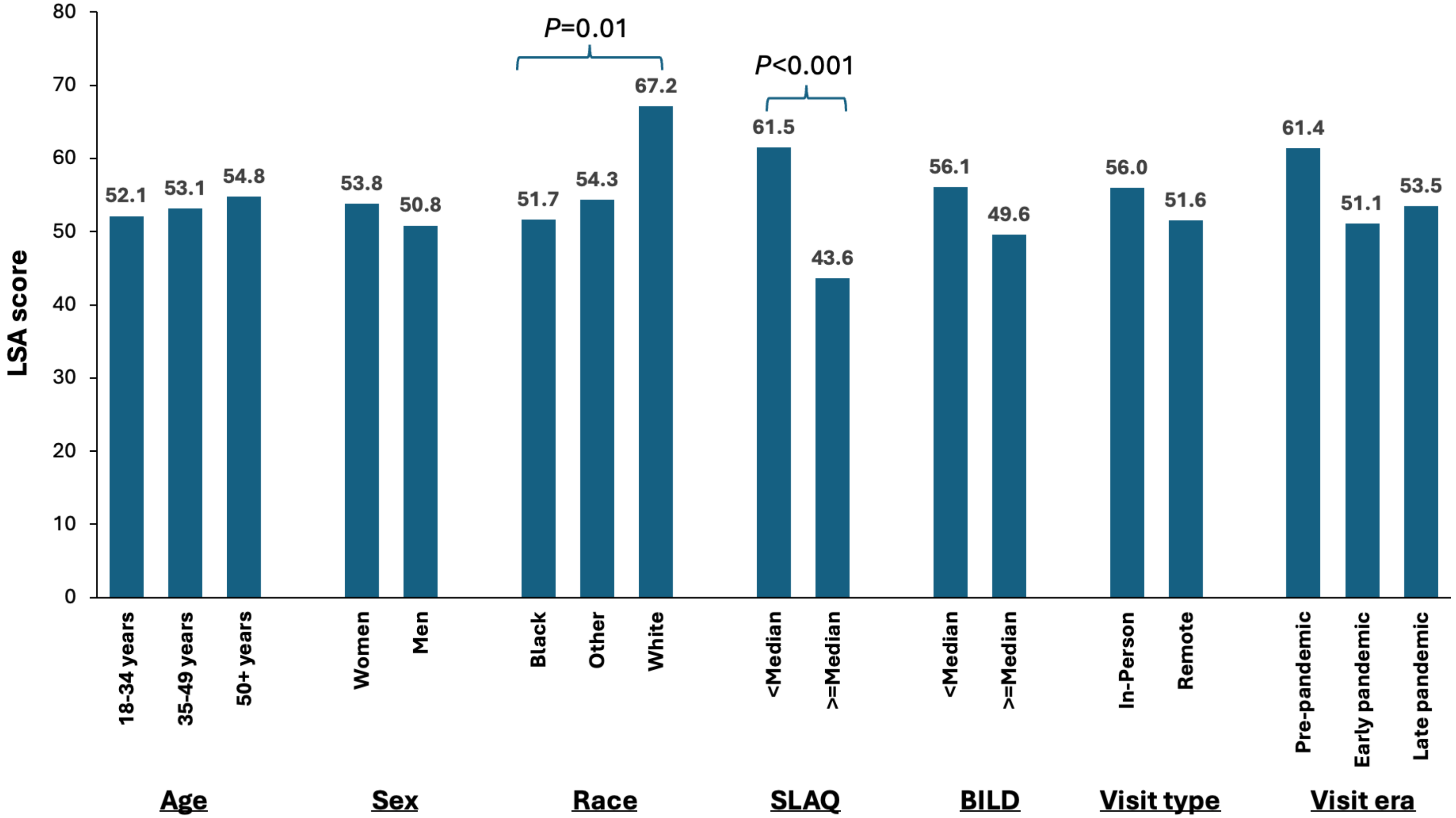

Table S2. Sensitivity analyses: associations of selected participant characteristics with additional adjustment for depressive symptoms; with the cutoff defined as any restriction in community mobility; and using continuous scores

	Fully Adjusted[a] Estimate (95% CI)				
	Percentage with restricted community mobility				
**Characteristic**	**Primary analysis**	**Additionally adjusted for depressive symptoms**	**Excluding work variable**	**Defined as any restrictions**	**Mean LSA score**
Age					
18-34 years	41.0 (31.1-50.8)	40.7 (30.7-50.7)	42.5 (32.9-52.2)	72.3 (63.1-81.4)	52.4 (45.6-59.2)
35-49 years	40.4 (33.4-47.3)	39.0 (31.9-46.0)	41.9 (35.0-48.8)	69.6 (62.8-76.3)	54.4 (49.6-59.3)
≥50 years (ref.)	41.2 (34.5-48.1)	42.2 (35.2-49.1)	41.4 (34.6-48.2)	77.9 (72.0-83.9)	53.3 (48.6-58.0)
Sex					
Women (ref.)	40.7 (36.2-45.3)	40.4 (35.8-45.0)	41.6 (37.1-46.1)	73.6 (69.4-77.8)	53.8 (50.7-57.0)
Men	42.4 (26.7-58.1)	43.3 (26.9-59.8)	44.1 (28.4-59.8)	72.7 (58.4-87.0)	50.6 (39.9-61.4)
Race					
Black (ref.)	43.4 (38.5-48.2)	43.4 (38.4-48.3)	44.5 (39.7-49.3)	74.9 (70.5-79.2)	51.9 (48.6-55.2)
Other	36.0 (18.4-53.6)	36.0 (18.4-53.6)	34.6 (17.6-51.6)	68.0 (50.6-85.4)	54.4 (42.0-66.7)
White	24.4 (12.7-36.2)	22.7 (11.1-34.4)	25.5 (13.8-37.2)	66.7 (53.5-79.8)	66.0 (56.8-75.2)
Education					
≤High school graduate	51.1 (41.4-60.7)[b]	50.0 (40.0-60.1)[b]	52.6 (43.0-62.1)	88.2 (81.8-94.7)[c]	42.4 (36.0-48.7)[b]
Some college	48.8 (41.4-56.1)[b]	49.0 (41.6-56.5)[b]	49.7 (42.5-56.9)	75.6 (69.1-82.1)	50.5 (45.6-55.3)
≥College graduate (ref.)	27.2 (20.7-33.6)	27.5 (20.9-34.1)	27.7 (21.2-34.2)	63.0 (55.7-70.2)	63.2 (58.3-68.0)
Currently working					
Yes	30.5 (24.4-36.5)	31.1 (24.9-37.3)	30.5 (24.4-36.5)	66.5 (60.1-72.9)	62.9 (58.5-67.3)
No (ref.)	50.2 (44.0-56.5)	49.5 (43.1-55.9)	50.2 (44.0-56.5)	79.9 (74.8-85.0)	45.2 (41.0-49.3)
Household annual income[d]					
<$30,000	55.3 (47.5-63.1)	54.2 (46.1-62.3)	57.4 (49.8-65.0)	84.4 (78.6-90.2)	41.3 (36.1-46.6)
$30,000-$69,999	36.1 (29.2-43.0)	36.8 (29.8-43.8)	36.3 (29.4-43.2)	68.1 (61.3-74.8)	57.0 (52.2-61.8)
≥$70,000 (ref.)	30.6 (22.1-39.1)	30.9 (22.4-39.5)	30.7 (22.2-39.2)	68.4 (59.4-77.3)	63.6 (57.3-69.9)
Receiving disability support[d]					
Yes	52.9 (46.1-59.7)	52.2 (45.3-59.2)	54.1 (47.5-60.8)	79.7 (74.1-85.2)	45.1 (40.6-49.6)

	Fully Adjusted[a] Estimate (95% CI)				
	Percentage with restricted community mobility				
**Characteristic**	**Primary analysis**	**Additionally adjusted for depressive symptoms**	**Excluding work variable**	**Defined as any restrictions**	**Mean LSA score**
No (ref.)	31.0 (25.3-36.7)	31.2 (25.4-37.0)	31.3 (25.6-37.0)	69.0 (63.2-74.8)	60.5 (56.4-64.6)
Disease activity					
SLAQ score<median (ref.)	28.5 (22.9-34.3)	28.7 (22.9-34.5)	29.7 (24.1-35.4)	68.3 (62.4-74.2)	61.8 (57.6-66.0)
SLAQ score≥median	55.2 (48.4-62.0)[c]	54.6 (47.7-61.5)[b]	56.1 (46.5-62.7)	79.7 (74.1-85.2)	44.0 (39.5-48.5)[c]
Disease damage					
BILD score<median (ref.)	38.3 (32.8-43.8)	38.3 (32.7-43.9)	38.9 (33.4-44.4)	69.9 (64.5-75.3)	56.2 (52.4-60.1)
BILD score≥median	45.0 (37.8-52.2)	44.4 (37.1-51.8)	46.4 (39.5-53.3)	79.4 (73.3-85.4)	49.4 (44.5-54.2)
Current steroids					
Yes (ref.)	45.7 (38.7-52.6)	44.9 (37.9-51.9)	47.2 (40.3-54.1)	76.8 (70.9-82.9)	49.6 (44.9-54.3)
No	37.2 (31.6-42.8)	37.3 (31.6-43.1)	37.7 (32.2-43.3)	71.1 (65.6-76.5)	56.5 (52.6-60.5)
Obese					
Yes	40.0 (33.6-46.4)	39.9 (33.4-46.4)	41.0 (34.6-47.4)	72.8 (66.9-78.8)	55.4 (50.9-59.8)
No (ref.)	42.2 (36.1-48.3)	42.2 (35.9-48.4)	43.1 (37.1-49.2)	74.9 (69.3-80.4)	51.8 (47.6-56.1)
Depressive symptoms					
PROMIS score<median (ref.)	33.6 (27.8-39.5)	33.6 (27.8-39.5)	34.9 (29.1-40.7)	70.0 (64.2-75.7)	58.6 (54.5-62.8)
PROMIS score≥median	49.4 (42.6-56.3)	49.4 (42.6-56.3)	50.3 (43.5-57.1)	77.5 (71.6-83.4)	47.5 (42.8-52.2)
Perceived stress					
PSS score<median (ref.)	33.2 (27.2-39.1)	33.0 (26.9-39.1)	33.6 (27.7-39.6)	68.2 (62.2-74.3)	60.2 (55.9-64.4)
PSS score≥median	47.8 (40.9-54.6)	48.0 (41.1-54.9)	48.9 (42.1-55.7)	77.0 (71.0-82.9)	47.7 (43.2-52.3)

BILD, Brief Index of Lupus Damage; BMI, body mass index; SLAQ, Systemic Lupus Activity Questionnaire; PROMIS, Patient Reported Outcomes Measurement Information System (Depression Short Form-8a); PSS, Perceived Stress Scale; UAB LSA, University of Alabama Birmingham Life-Space Assessment.

[a]Adjusted for: continuous age, sex, race, education, work status, and continuous SLAQ and BILD scores.

[b]P<0.05 vs. referent, from logistic regression.

[c]P<0.001 vs. referent, from logistic regression.

[d]From the closest Georgians Organized Against Lupus (GOAL; parent study) assessment.

Table S3. Sensitivity analyses: associations of selected participant characteristics, stratified by type and era of study visit

	Unadjusted marginal percentages for reaching neighborhood level weekly and independently (95% CI)				
	Type of visit		Pandemic era		
Characteristic	In-person	Remote	Pre-pandemic	Early pandemic	Late pandemic
Age					
18-34 years	34.1 (20.1-48.1)	48.9 (34.6-63.2)	11.1 (-9.4-31.6)	33.3 (14.4-52.2)	50.0 (37.1-62.9)
35-49 years	43.4 (32.3-54.6)	43.3 (33.4-53.2)	53.8 (26.7-80.9)	52.5 (39.8-65.3)	36.6 (27.2-46.0)
≥50 years	40.5 (30.0-51.0)	39.4 (29.8-49.0)	32.1 (14.8-49.4)	43.8 (31.6-55.9)	39.6 (29.5-49.6)
Sex					
Women	40.2 (33.0-47.4)	42.9 (36.5-49.2)	38.1 (23.4-35.4)	45.7 (6.2-79.5)	40.0 (33.6-46.3)
Men	40.0 (20.8-59.2)	41.7 (13.8-69.6)	12.5 (-10.4-35.4)	42.9 (37.5-54.0)	50.0 (29.1-70.9)
Race					
Black	42.4 (35.1-49.7)	46.4 (39.2-53.4)	35.6 (21.6-49.5)	50.5 (41.1-59.8)	43.3 (36.6-49.9)
Other	16.7 (-4.4-37.7)	53.3 (28.1-78.6)	---	60.0 (29.6-90.4)	26.7 (4.3-49.0)
White	33.3 (9.5-57.2)	19.4 (6.5-32.4)	33.3 (-20.0-86.7)	21.4 (6.2-36.6)	25.0 (6.0-44.0)
Education					
≤High school graduate	53.4 (40.6-66.3)	53.3 (38.8-67.9)	43.8 (19.4-68.1)	61.5 (42.8-80.2)	52.5 (39.9-65.0)
Some college	47.1 (35.4-58.8)	51.0 (41.2-60.8)	43.8 (19.4-68.1)	49.2 (37.1-61.4)	50.6 (40.2-60.9)
≥College graduate	23.7 (14.1-33.2)	29.6 (20.6-38.6)	16.7 (-0.5-33.9)	33.9 (21.5-46.3)	25.0 (16.5-33.5)
Currently working					
Yes	28.2 (18.7-37.8)	31.7 (23.3-40.0)	23.5 (3.4-43.7)	34.3 (23.0-45.7)	28.9 (20.8-37.0)
No	46.8 (37.5-56.1)	52.9 (44.0-61.9)	37.5 (20.7-54.3)	55.1 (44.1-66.2)	50.0 (41.1-58.9)
Household annual income[b]					
<$30,000	57.3 (46.6-68.0)	58.3 (46.9-69.7)	54.2 (34.2-74.1)	65.9 (51.9-79.9)	54.7 (44.1-65.2)
$30,000-$69,999	29.1 (19.1-39.1)	40.9 (30.9-50.9)	11.1 (-3.4-25.6)	42.9 (29.9-55.8)	35.7 (26.2-45.2)
≥$70,000	32.4 (17.3-47.5)	28.2 (17.7-38.6)	40.0 (-2.9-82.9)	26.2 (12.9-39.5)	31.1 (19.5-42.8)
Receiving disability support[b]					
Yes	51.0 (41.1-60.9)	55.8 (46.2-65.3)	36.4 (20.0-52.8)	60.3 (48.7-71.9)	54.5 (44.7-64.2)
No	30.5 (21.7-39.3)	32.4 (24.5-40.2)	31.3 (8.6-54.0)	33.3 (22.9-43.8)	30.6 (23.2-38.1)
Disease activity					

	Unadjusted marginal percentages for reaching neighborhood level weekly and independently (95% CI)				
	Type of visit		Pandemic era		
**Characteristic**	**In-person**	**Remote**	**Pre-pandemic**	**Early pandemic**	**Late pandemic**
SLAQ score<median	25.5 (16.9-34.1)	32.8 (24.9-40.9)	18.2 (2.1-34.3)	36.6 (26.2-47.0)	27.3 (19.6-35.1)
SLAQ score≥median	56.1 (46.3-65.9)	56.1 (46.3-65.9)	50.0 (30.8-69.2)	57.9 (45.1-70.7)	56.6 (47.5-65.8)
Disease damage					
BILD score<median	40.9 (32.4-49.5)	38.3 (30.5-46.1)	38.2 (21.9-54.6)	39.5 (29.2-49.9)	39.7 (32.1-47.4)
BILD score≥median	39.0 (28.1-49.9)	50.0 (39.9-60.1)	25.0 (3.8-46.2)	54.1 (41.6-66.6)	42.6 (32.6-52.5)
Current steroids					
Yes	43.5 (32.6-54.6)	48.7 (39.5-57.9)	38.1 (17.3-58.9)	50.7 (38.8-62.7)	45.6 (36.0-55.3)
No	37.6 (29.1-46.1)	37.6 (29.4-46.0)	31.0 (14.2-47.9)	41.3 (30.5-52.0)	37.0 (29.2-44.8)
Depressive symptoms					
PROMIS score<median	33.0 (23.8-42.2)	36.8 (28.6-45.0)	17.4 (1.9-32.9)	40.5 (29.7-51.3)	35.1 (26.9-43.3)
PROMIS score≥median	46.9 (36.9-56.9)	51.5 (41.7-61.2)	45.8 (25.9-65.8)	51.6 (39.2-64.1)	48.6 (39.4-57.9)
Perceived stress					
PSS score<median	32.3 (23.1-41.5)	34.4 (26.1-42.7)	16.0 (1.6-30.4)	36.1 (25.8-46.5)	35.3 (26.6-44.0)
PSS score≥median	49.4 (38.9-59.9)	49.0 (39.2-58.7)	50.0 (29.1-70.9)	55.2 (42.4-68.0)	45.9 (36.5-55.2)

P for interaction >0.05 for all models. BILD, Brief Index of Lupus Damage; BMI, body mass index; SLAQ, Systemic Lupus Activity Questionnaire; PROMIS, Patient Reported Outcomes Measurement Information System (Depression Short Form-8a); PSS, Perceived Stress Scale; UAB LSA, University of Alabama Birmingham Life-Space Assessment.

[a]Adjusted for: continuous age, sex, race, education, work status, and continuous SLAQ and BILD scores.

[b]From the closest Georgians Organized Against Lupus (GOAL; parent study) assessment.
